# Clinical outcomes in patients admitted to a hospitalist service exposed to an antimicrobial stewardship program – a retrospective matched cohort study

**DOI:** 10.1186/s13756-019-0542-2

**Published:** 2019-05-24

**Authors:** E. Rennert-May, J. Conly, G. Chen, B. Dalton

**Affiliations:** 1 Department of Medicine, Cumming School of Medicine, University of Calgary, and Alberta Health Services, AGW5 Ground Floor SSB, Foothills Medical Centre, 1403 29 St NW, Calgary, AB T2N 2T9 Canada; 2 Department of Medicine, Department of Pathology & Laboratory Medicine, Department of Microbiology, Immunology and Infectious Diseases, Cumming School of Medicine, University of Calgary, Alberta Health Services, O’Brien Institute for Public Health, and Snyder Institute for Chronic Diseases, Calgary, AB Canada; 3 Community Health Sciences, Cumming School of Medicine, University of Calgary and Research Facilitation, Alberta Health Services, Calgary, AB Canada; 4 Pharmacy Services, Alberta Health Services, and O’Brien Institute for Public Health, Calgary, AB Canada

**Keywords:** Antimicrobial stewardship, Antibiotic resistance, Antimicrobial stewardship interventions

## Abstract

**Background:**

Given global issues with antimicrobial resistance and a need to optimize antimicrobial usage, antimicrobial stewardship (AS) programs are becoming a necessary component of hospitals and are increasingly mandated worldwide. It is important to evaluate these programs with respect to relevant clinical outcomes.

**Methods:**

An AS program with a prospective audit and feedback service (PAF) of antimicrobial usage was initiated May 11, 2015 at our tertiary care center, for patients admitted under the hospitalist service. We conducted a retrospective matched cohort study. Patients assessed during the first year of this PAF were considered to be the exposed cohort and were compared to unexposed controls matched on gender, age and infectious diagnosis selected from patients who had been admitted under the hospitalist service prior to initiation of the PAF. Descriptive analysis was completed and a multivariate conditional logistic regression was performed to analyze differences between the exposed and control groups in terms of a composite endpoint of 30 day mortality, 30 day post hospital discharge mortality and hospital re-admission.

**Results:**

A total of 348 patients were assessed and received PAF suggestions during the first year were compared to 827 matched control patients who did not receive PAF suggestions. Of 707 PAF suggestions made, the most common was to stop an antimicrobial (23%). A significantly lower (20.7% vs 28.8%, *p* = 0.008) composite endpoint was found in the group exposed to the PAF (OR 0.71 95%CI 0.52–0.97). This difference persisted when only patients with PAF suggestions that were completely or partially accepted were considered (18.6% vs 28.5%, *p* = 0.001) but was no longer significant when patients who had their ASP suggestions declined were analyzed (30.2% vs 26.7%, *p* = 0.610).

**Conclusions:**

In this retrospective cohort study, patient admissions in which PAF recommendations were accepted had better clinical outcomes than matched historical controls managed in the absence of this AS service.

## Background

The World Health Organization (WHO), Infectious Diseases Society of America (IDSA) and Centers for Disease Control and Prevention (CDC) have recognized antimicrobial resistance as a global health crisis [1–3]. High intensity use of antimicrobials in hospitals creates an environment that selects for resistant organisms [4–9].

An antimicrobial stewardship (AS) program has been in place at our tertiary care hospital site since 2011, with increasing elements over time. The AS program currently includes formulary review with prescribing regulation through guidelines, therapeutic substitutions and restrictions, as well as educational initiatives and surveillance of antimicrobial utilization. Our local jurisdiction has also been active in other antimicrobial stewardship (AS) initiatives including the creation and implementation of electronic order-sets to promote appropriate use of antimicrobials and ensure high-quality patient care, education of trainees including an AS preceptorship program for pharmacists, and quality improvement initiatives.

Several multi-faceted measures have been used to assess the efficacy and acceptability of our site’s AS prospective audit and feedback service (PAF) including number of patients assessed, acceptance of suggestions, and whether or not the supporting infectious diseases physician was involved. In addition, antimicrobial utilization for the hospitalist service was found to be reduced after introduction of the PAF by segmented regression [10]. The increasing evidence of the benefit of AS programs on intended outcomes such as reduced antimicrobial usage, and clinical outcome measures including hospital length of stay and mortality, has been described in recent systematic reviews [11, 12]. In one recent systematic review, mortality in response to different AS interventions was examined [11]. Guideline concordant empirical antibiotic therapy and de-escalation of therapy had a significant effect on mortality though there was substantial heterogeneity in the included studies [11]. Non-significant reductions in mortality were noted in studies that examined switching from intravenous to oral therapy, therapeutic drug monitoring and restriction of antibiotics as well as bedside consultations [11]. A recent Cochrane review found that lower antibiotic use likely does not increase mortality [13]. However, there is still a lack of robust and high quality evidence on clinical outcomes [11]. There is a need for ongoing evaluation to assess outcomes of patients with respect to any potential negative impact that suggestions by an AS team might cause.

We therefore sought to determine if a change in mortality and hospital re-admission rates was observable in hospitalist patients who were reviewed by our site’s PAF and for whom antimicrobial recommendations were made.

## Methods

### Setting and patient population

The study setting was an 1100 bed urban tertiary care hospital where there are 2.0 full-time equivalent (FTE) pharmacist positions dedicated to AS. A PAF for the hospitalist units was established for hospitalist patients May 11, 2015. PAF has been recognized as a successful AS intervention [14] and the hospitalist physicians had identified the need for a quality improvement initiative for antimicrobial use internally.

The hospitalist service is a general medicine service that admits adult patients (i.e. > 18 years of age) with typically a single system active disease, usually for short admissions, and for which infections are a common primary or secondary diagnosis. Although many patients have multiple complex comorbid conditions, patients are typically less complex than those admitted under the internal medicine service. There are approximately 200 patients on any given day under the care of the hospitalist physicians. The hospital site for this initiative has a computerized physician order entry (CPOE) system (All Scripts version 16.3). Therefore, information technology support was sought to develop a daily report of all newly admitted patients under the hospitalist service including details of antimicrobial orders, to identify target patients for PAF suggestions. The CPOE system is a comprehensive electronic patient care management system that includes additional clinical information on labs, vital signs, physician and other health care professional notes.

Pharmacists with experience on the Infectious Diseases (ID) consult service were scheduled to dedicate time to review newly admitted patients on antimicrobial therapy on the hospitalist service (weekdays only excluding statutory holidays). An ID physician was available to review assessments and suggestions at the discretion of the pharmacist. A database (Microsoft Excel V15.26, Redmond, Washington) of patients for whom PAF suggestions were made was maintained by the pharmacists, and patients were reviewed in the days following the suggestions to determine if suggestions were accepted, partially accepted or declined [10].

Communication of the assessments and suggestions by the PAF pharmacists were in the form of multidisciplinary progress reports in the CPOE system. These notes were also printed and placed within the physician progress notes of the paper chart to ensure they were read.

### Study design

For the current study, a retrospective matched cohort was conducted. Patients reviewed by PAF for whom suggestions were made in the first year of program implementation (May 11, 2015 to May 11, 2016) were considered the exposed cohort. The unexposed cohort of patients was selected from patients under hospitalist care before PAF was initiated (from May 1, 2014 to April 30, 2015). Up to three unexposed control patients per exposed patient were selected with matching on the basis of gender, age +/− 5 years and infectious diagnosis(es) from medical records coding. For the hospitalist service, diagnoses of pneumonia and urinary tract infection are common and represented 78% of patients assessed in the first year of the program by AS pharmacist records. Since other types of infections were uncommon and infection coding was often incomplete, we grouped incomplete and other infections together. For study purposes the diagnosis was qualified by whether the infection was a primary or secondary diagnosis. If more than three unexposed controls were available, three were chosen using the random number generator function on Excel (Microsoft Excel V15.26, Redmond, Washington). The unexposed control cohort patients could only be selected once and the exposed cohort patients who had been admitted during the previous year were excluded from the control pool to avoid the same patient being in the study more than once. Selection of unexposed controls was prioritized by order of perceived higher mortality/morbidity (e.g. primary diagnosis pneumonia would be first, primary diagnosis of urinary tract infection second, secondary diagnosis of pneumonia third).

### Outcomes

The primary outcome measure was a composite endpoint of 30 day mortality, 30 day post hospital discharge mortality, and 30 day hospital re-admission rates.

### Statistical analysis

Descriptive analysis of the comparison groups was performed using student’s t-test for continuous variables and Chi square test for categorical variables. Multivariate conditional logistic regression was used to determine the differences between the exposed and unexposed cohorts for the primary outcome. The difference between the exposed and unexposed cohorts was also estimated after adjusting for age, gender, and Charlson comorbidities score [15]. Charlson comorbidity scores were grouped as 0, 1–2, and > 3 in the model. Statistical significance at *p* < 0.10 level in the analysis was deemed sufficiently suggestive of its importance to include in multivariate models. Sub-analyses by suggestion acceptance rating was performed by full acceptance, partial acceptance [defined as the most responsible physician choosing a duration of antimicrobial therapy that was 1–2 days more than the duration suggested by the PAF, or an alternative antimicrobial albeit with a similar spectrum instead of the agent suggested, or in addition to other recommendations an ID consultation was recommended but not acted upon] and non-acceptance. All statistical analyses were performed using SAS software version 9.4 (SAS Institute, Cary NC).

Review and approval of the research protocol was undertaken by the Conjoint Health Research Ethics Board at the University of Calgary (REB17–1369, November 28, 2017).

## Results

**Fig. 1:**
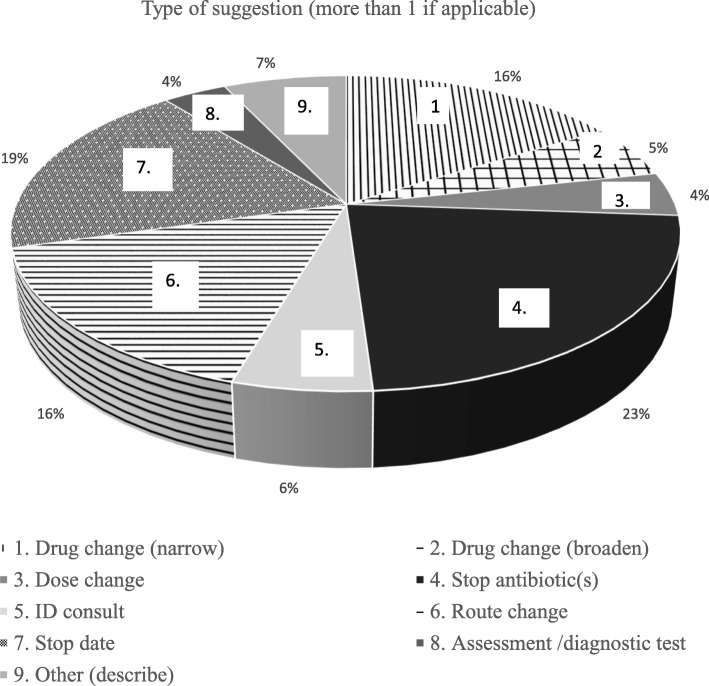
Type of Suggestion Offered by the AS Service May 2015-Apr 2016 (Inclusive). Abbreviations: AS = Antimicrobial Stewardship, ID = Infectious Diseases

The demographic characteristics seen in Table 1 demonstrate that there was no significant difference between the exposed group and matched cohort for age, gender, and secondary infectious diagnosis. However, there was a significant difference between the two groups in terms of the three categories of Charlson Comorbidity Index (*p* = 0.001) and one of four categories of primary infectious diagnosis (*p* = 0.012) with higher scores for each in the unexposed cohort (see Table 1). A total of 707 suggestions were made and the distribution of recommendations is displayed in Fig. 1. The most common suggestion made by the PAF was to stop an antimicrobial (163, [23%]). The next most common recommendations were to adjust the stop date of an antimicrobial (134, [19%]), narrow an antibiotic (113, [16%]), and alter the route of administration (113, [16%]).

**Table 1:** Characteristics of the study cohort exposed to the PAF and unexposed matched controls. May 2015–April 2016

total – n (%)	PAF exposed group	Matched unexposed controls	*p* value
	348 (100)	827 (100)	
age group			
< 35	12 (3.4)	26 (3.1)	0.961
35–55	29 (8.3)	68 (8.2)	
55–65	37 (10.6)	81 (9.8)	
> = 65	270 (77.6)	652 (78.8)	
Gender			
Female	190 (54.6)	431 (52.1)	0.437
Male	158 (45.4)	396 (47.9)	
Infectious Diagnosis			
Primary diagnosis			
Primary diagnosis of pneumonia and secondary diagnosis of UTI	4 (1.1)	6 (0.7)	0.012
Primary diagnosis of pneumonia	26 (7.5)	72 (8.7)	
Primary diagnosis of UTI	37 (10.6)	45 (5.4)	
Lack of primary diagnosis of infection	281 (80.7)	704 (85.1)	
Secondary diagnosis			
Secondary diagnosis of pneumonia and UTI	9 (2.6)	22 (2.7)	0.903
Secondary diagnosis of pneumonia	48 (13.8)	127 (15.4)	
Secondary diagnosis of UTI	79 (22.7)	178 (21.5)	
Lack of secondary diagnosis of infection	212 (60.9)	500 (60.5)	
Charlson Comorbidity Score			
0	252 (72.4)	507 (61.3)	0.001
1 or 2	75 (21.6)	230 (27.8)	
> 3	21 (6)	90 (10.9)	

*Abbreviations*: *PAF* Prospective audit and feedback service, *UTI* Urinary Tract Infection

In total, 348 patients were assessed and had subsequent suggestions regarding antimicrobial therapy during the first year of the hospitalist PAF. An average of 2.4 controls were matched (827 total controls) to each exposed patient. Acceptance of suggestions was high with 66.1% (467/707) recorded as fully accepted, 15.8% (112/707) partially accepted and 18.1% (128/707) as non-accepted.

Unadjusted analysis of patients reaching clinical endpoints in the study cohort and matched controls are presented in Table 2. The composite endpoint was significantly less likely to occur in the exposed (20.7%) versus the unexposed (28.2%) cohort overall (*p* = 0.008). This finding was also observed when only patients who had suggestions from the PAF fully or partially accepted were considered compared to those who had suggestions declined (18.6% vs 28.5%, respectively, *p* = 0.001). The difference was no longer significant when only patients with declined suggestions from the PAF were analyzed (30.2% vs 26.7%, respectively, *p* = 0.610). Adjusted analyses from the multivariate conditional logistic regression are presented in Table 3. Patients who were in the exposed versus the unexposed cohort were less likely to be associated with the composite endpoint (OR 0.71 95%CI 0.52–0.97). This observation remained when only exposed cohort patients with partial or full suggestion acceptance were considered (OR 0.61 95%CI 0.43–0.87), but not when exposed cohort patients with declined suggestions were analyzed (OR 1.26 95%CI 0.66–2.40). Patients were more likely to be associated with the composite endpoint when they had a Charlson comorbidity score of 1–2 versus 0 (OR 1.78 95%CI 1.22–2.59) and > 3 versus 0 (OR 1.96 95%CI 1.18–2.26).

**Table 2:** Unadjusted analysis of study cohorts exposed to the PAF and unexposed matched controls. All patients and grouping by suggestion acceptance

	Exposed cohort	Unexposed cohort	*p* value
All Patients			
total - n (%)	348 (100)	827 (100)	
Composite Endpoint	72 (20.7)	233 (28.2)	0.008
re-admission within 30 days^a^	37 (11.8)	76 (11.3)	
mortality in hospital within 30 days	35 (11.3)	157 (20.9)	
30 day mortality post discharge^a^	10 (3.5)	71 (10.7)	
No endpoint reached	276 (79.3)	594 (71.8)	
PAF Cohort - Suggestion Accepted or Partial Acceptance			
total - n (%)	285 (100)	681 (100)	
Composite Endpoint	53 (18.6)	194 (28.5)	0.001
re-admission within 30 days^a^	32 (12.1)	65 (11.8)	
mortality in hospital within 30 days	21 (8.3)	129 (20.9)	
30 day mortality post discharge^a^	5 (2.1)	57 (10.5)	
No endpoint reached	232 (81.4)	487 (71.5)	
PAF Cohort - Suggestion Declined			
total - n (%)	63 (100)	146 (100)	
Composite Endpoint	19 (30.2)	39 (26.7)	0.610
re-admission within 30 days^a^	5 (10.2)	11 (9.3)	
mortality in hospital within 30 days	14 (24.1)	28 (20.7)	
30 day mortality post discharge^a^	5 (10.2)	14 (11.6)	
No endpoint reached	44 (69.8)	107 (73.3)	

*Abbreviations*: *PAF* Prospective audit and feedback service

^a^Some patients met two composite endpoint criteria, (i.e., 30-day readmission and 30-day post discharge mortality), therefore of all addition of individual components may be greater than total composite endpoint

**Table 3:** Multivariate logistic regression models showing co factor association with composite outcome (odds ratios with 95% confidence intervals) related to all suggestions and grouped according to suggestion acceptance

All Patients		
Variable	age (per year)	1.03 (0.97–1.10)
	Female vs Male	1.34 (0.96–1.87)
	PAF versus Control Pt	0.71 (0.52–0.97)
	Charlson 1–2 vs 0	1.78 (1.22–2.59)
	Charlson > = 3 vs 0	1.96 (1.18–3.26)
Suggestion Accepted or Partial Acceptance		
Variable	age (per year)	1.04 (0.97–1.12)
	Female vs Male	1.26 (0.87–1.83)
	PAF versus Control Pt	0.61 (0.43–0.87)
	Charlson 1–2 vs 0	1.73 (1.14–2.63)
	Charlson > = 3 vs 0	2.29 (1.29–4.04)
Suggestion Declined		
Variable	age (per year)	1.01 (0.87–1.17)
	Female vs Male	1.77 (0.81–3.85)
	PAF versus Control Pt	1.26 (0.66–2.40)
	Charlson 1–2 vs 0	2.15 (0.89–5.20)
	Charlson > = 3 vs 0	0.98 (0.29–3.27)

*Abbreviations*: *PAF* Prospective audit and feedback service, *Pt* Patient

## Discussion

AS programs have been found to improve antimicrobial prescribing and reduce overall antimicrobial utilization, thus reducing antimicrobial pressure in the hospital environment that can drive antimicrobial resistance. When such programs involve direct advice to the treating clinicians, it is of utmost importance that no harm results to patients. Our results compliment other published studies that documented clinical outcomes before and after introduction of ASP [16–22]. Few of these studies focussed on such outcomes and therefore quality of analysis of outcomes varies considerably. We feel our focus on this important aspect of ASPs is a valuable addition to the body of literature on stewardship.

Our primary outcome was the composite endpoint of hospital re-admission and 30 day in hospital or post discharge mortality which was seen significantly less often in the exposed cohort. When only the exposed cohort who had partial or complete acceptance of suggestions by the PAF was considered, this significant difference was the same or greater. This latter observation within our hospitalist population supports a relationship between the PAF intervention and the composite outcome.

Both the unadjusted differences and the regression model results supported the association of lower mortality and hospital re-admissions in the cohort who received PAF suggestions. At the very least, these findings provide reassurance that the PAF did not contribute to adverse outcomes. Patients with a higher Charlson Comorbidity score may be expected to be associated with the composite endpoint more often as a greater number of comorbidities are generally found in sicker patients who would be more likely to die or need frequent hospital admissions [15, 23]. It is important to note that the exposed cohort had a greater quantity of patients with a Charlson comorbidity score of 0 (72.4%) compared to the unexposed cohort (61.3%). This could have created a bias as patients with fewer comorbidities may have been less likely to be associated with the composite endpoint. However, if this were the case the difference in composite endpoints should have persisted between the exposed and unexposed cohorts whether or not AS suggestions were accepted, which was not demonstrated (data not shown).

Our findings are consistent with a 2017 Cochrane review that found that AS interventions that improve antimicrobial prescribing in the setting of hospital inpatients can safely decrease the length of antimicrobial therapy and do not increase mortality [13]. Our observation that a PAF does not result in decreased cure rates is consistent with previous findings [24].

The impact of AS programs on patient clinical outcomes was assessed recently as discussed above [11]. Both of the elements associated with reduced mortality, i.e. guideline adherent therapy and de-escalation of therapy, are achieved through a PAF intervention which was utilized in the current study, and our results are consistent with the findings from the systematic review.

Our study adds to the literature as there are few studies done on AS interventions in patients admitted to hospital under a hospitalist service. Additionally, while there is a previous study that used propensity score matching in the evaluation of a PAF [25], there are no studies that we know of that used a retrospective matched cohort design to evaluate a PAF. A strength of our study was that it allowed for the evaluation of clinical outcomes of a PAF, as opposed to process outcomes which are more commonly examined. It is of critical importance to monitor the outcomes of different AS programs and interventions and ensure there are no adverse events, which was demonstrated in our study. Additionally, while this AS program is a hospital based program, many hospitalist physicians also work in the community and practice outpatient medicine. The positive findings from the current study support the use of PAF which will aid in encouraging hospitalist physicians to apply best practices for optimizing antimicrobial care in their outpatient settings and support outpatient AS programs.

We acknowledge the limitations of our study. The use of a historic rather than a contemporaneous control patient population may have introduced bias and confounding factors related to the comparability of the groups. However, the patients were from the same catchment area and admitted to the same service and wards with no major changes in attending physicians. Although all patients in the two cohorts received antibiotics, the medical record coding data was lacking an infectious diagnosis in approximately 60% of cases in both groups. However, we did not detect any major imbalance or bias favouring one group over the other. In addition the use of matching by age (+/− 5 years) and matching up to 3 controls per exposed patients would mitigate the risk of confounding as variance would be expected to be less due to the larger matched sample size [26].We did not have access to a severity of illness score which may be a limitation although we were able to adjust by Charlson comorbidity score which is a measure of chronic illness burden [15] Despite our mitigating measures, we acknowledge that an observational study design cannot prove a causal relationship and other factors may have led to the observed findings. Therefore the observed difference in composite endpoint should be interpreted within the context of this limitation. Finally, the composite endpoint was only considered to 30 days. Longer follow up should be considered to ensure there are no adverse events that are not immediately apparent through the use of PAF.

Future studies should consider a comparison of the composite endpoints in patients under our hospitalist service to patients on hospitalist services in other hospitals with no AS programs to negate the need for historic controls. Previous literature suggests that AS programs are cost-effective [12] and so, further studies should consider the cost-effectiveness of our AS program as it would provide a unique Canadian perspective.

## Conclusions

Ultimately the goal of AS programs is to improve patient safety and quality of care. We were able to demonstrate that there was no increase in adverse clinical outcomes as a result of an PAF and that there was a positive association with exposure to the PAF. Studies such as the current one are imperative as AS programs are becoming increasingly common and legislated in healthcare settings. It is necessary to demonstrate that PAF are not associated with negative patient outcomes. Future research should strive to establish the enhanced clinical benefits as a result of AS programs as well as their potential for cost savings.

## Abbreviations

AS: Antimicrobial Stewardship
CDC: Centers for Disease Control and Prevention
CPOE: Computerized Physician Order Entry
FTE: Full-time Equivalent
IDSA: Infectious Diseases Society of America
PAF: Prospective Audit and Feedback Service
WHO: World Health Organization
